# Genetic analysis for the grain number heterosis of a super-hybrid rice WFYT025 combination using RNA-Seq

**DOI:** 10.1186/s12284-018-0229-y

**Published:** 2018-06-15

**Authors:** Liang Chen, Jianmin Bian, Shilai Shi, Jianfeng Yu, Hira Khanzada, Ghulam Mustafa Wassan, Changlan Zhu, Xin Luo, Shan Tong, Xiaorong Yang, Xiaosong Peng, Shuang Yong, Qiuying Yu, Xiaopeng He, Junru Fu, Xiaorong Chen, Lifang Hu, Linjuan Ouyang, Haohua He

**Affiliations:** 1 Key Laboratory of Crop Physiology, Ecology and Genetic Breeding, Ministry of Education, Jiangxi Agricultural University, Nanchang, 330045 China; 2 College of Agronomy, Jiangxi Agricultural University, Nanchang, 330045 China; 3 Southern Regional Collaborative Innovation Center for Grain and Oil Crops in China, Changsha, China

**Keywords:** Rice, Super-hybrid Rice, Heterosis, Grain number, RNA-seq

## Abstract

**Background:**

Despite the great contributions of utilizing heterosis to crop productivity worldwide, the molecular mechanism of heterosis remains largely unexplored. Thus, the present research is focused on the grain number heterosis of a widely used late-cropping *indica* super hybrid rice combination in China using a high-throughput next-generation RNA-seq strategy.

**Results:**

Here, we obtained 872 million clean reads, and at least one read could maps 27,917 transcripts out of 35,679 annotations. Transcript differential expression analysis revealed a total of 5910 differentially expressed genes (DG_HP_) between super-hybrid rice Wufengyou T025 (WFYT025) and its parents were identified in the young panicles. Out of the 5910 DG_HP_, 63.1% had a genetic action mode of over-dominance, 17.3% had a complete-dominance action, 15.6% had a partial-dominance action and 4.0% had an additive action. DG_HP_ were significantly enriched in carotenoid biosynthesis, diterpenoid biosynthesis and plant hormone signal transduction pathways, with the key genes involved in the three pathways being up-regulated in the hybrid. By comparing the DG_HP_ enriched in the KEGG pathway with QTLs associated with grain number, several DG_HP_ were located on the same chromosomal segment with some of these grain number QTLs.

**Conclusion:**

Through young panicle development transcriptome analysis, we conclude that the over-dominant effect is probably the major contributor to the grain number heterosis of WFYT025. The DG_HP_ sharing the same location with grain number QTLs could be considered a candidate gene and provide valuable targets for the cloning and functional analysis of these grain number QTLs.

**Electronic supplementary material:**

The online version of this article (10.1186/s12284-018-0229-y) contains supplementary material, which is available to authorized users.

## Background

Heterosis is a phenomenon in which hybrids exhibit superiority over their parental lines in economic traits, such as enhanced biomass production, development rate, stress tolerance and, most important, grain yield. Heterosis has been extensively used to increase crop productivity throughout the world. A major increase in rice yield was caused by the application of heterosis. Because of the key role of heterosis, the molecular mechanisms should be elucidated. In the early twentieth century, dominance (Davenport 1908) and over-dominance (Shull 1908) were used to explain heterosis. However, with nothing about molecular concepts being covered, consequently, they cannot interpret the molecular genetic mechanisms of heterosis (Birchler et al. 2003). With the development of polymerase chain reaction (PCR), molecular markers have been widely used to identify the distance between the hybrid and its parents and to build the relationship between heterosis and genetic distance. However, marker PCR can only be used to classify heterotic groups and determine genetic diversity, but it cannot predict heterosis because the coefficient of the relationship between the genetic distance of SSR markers and yield heterosis is very small (Xu et al. 2009). Subsequently, molecular markers and hybrid genetic analysis have been used to locate QTLs for heterosis. A Pioneer study of the heterosis gene *qGY2–1* related to yield was reported in haplotype populations (He et al. 2006). To eliminate the epistasis effect among QTLs, Bian et al. (2011) used chromosome segment substitution lines (CSSLs) to study heterosis for yield traits in *indica × japonica* hybrid rice subspecies. With the advent of high-throughput sequencing technology, scientists conducted DNA sequencing of 1495 elite hybrid rice varieties and their inbred parental lines. Comprehensive analyses of heterozygous genotypes have revealed that heterosis mainly resulted from the accumulation of numerous superior alleles with positive dominant effects (Huang et al. 2015).

In addition, the association of heterosis with differentially expressed transcripts was also investigated at the RNA level. Wei et al. (2009) investigated differentially expressed transcripts from tissues at different growth development stages using super rice LYP9 and its parents and found that the differentially expressed transcripts were closely related to QTLs in response to heterosis. Huang et al. (2006) used 9198 unique sequence tags to study gene differential expression profiles of young panicles using the super rice SY63 combination and suggested that transcripts controlling DNA repair and replication were up-regulated and that the genes related to carbohydrate, energy and lipid metabolism, translation and protein degradation were down-regulated.

High-throughput RNA sequencing has been used to search for heterosis in rice to avoid defects of methods with low throughput, high cost, low sensitivity, clonal preference, and high background noise. RNA-seq was first used to compare the transcriptome profiles of reciprocal hybrids from Nipponbare and 93-11, along with their parents, at the seedling stage. In total, 2800 genes showed differential expression, and these transcripts were involved in energy metabolism, especially in the Calvin cycle, in which six key components were up-regulated (He et al. 2010). Later, Zhai et al. (2013) compared the transcriptome between super hybrid XY9308 and its parents through RNA-seq, which indicated that carbohydrate metabolism and plant hormone signal transduction were enriched in differentially expressed transcripts.

In this study, we focused on heterosis in the rice WFYT025, a widely used late-cropping *indica* super hybrid rice combination in China. The number of filled grains, one of the most important yield heteroses in yield contributing factors, showed great differences between WFYT025 and its female parent. Thus, we conducted transcriptome analysis using young panicles from the WFYT025 combination by high-throughput RNA-seq to detect the correlation of key transcripts with filled grain number heterosis. Some key transcripts were mapped in the QTL interval related to grain number. Revealing the function of these transcripts may provide useful information for understanding the molecular mechanism underlying heterosis.

## Results

### Phenotype analysis for WFYT025 and its parents

**Fig. 1:**
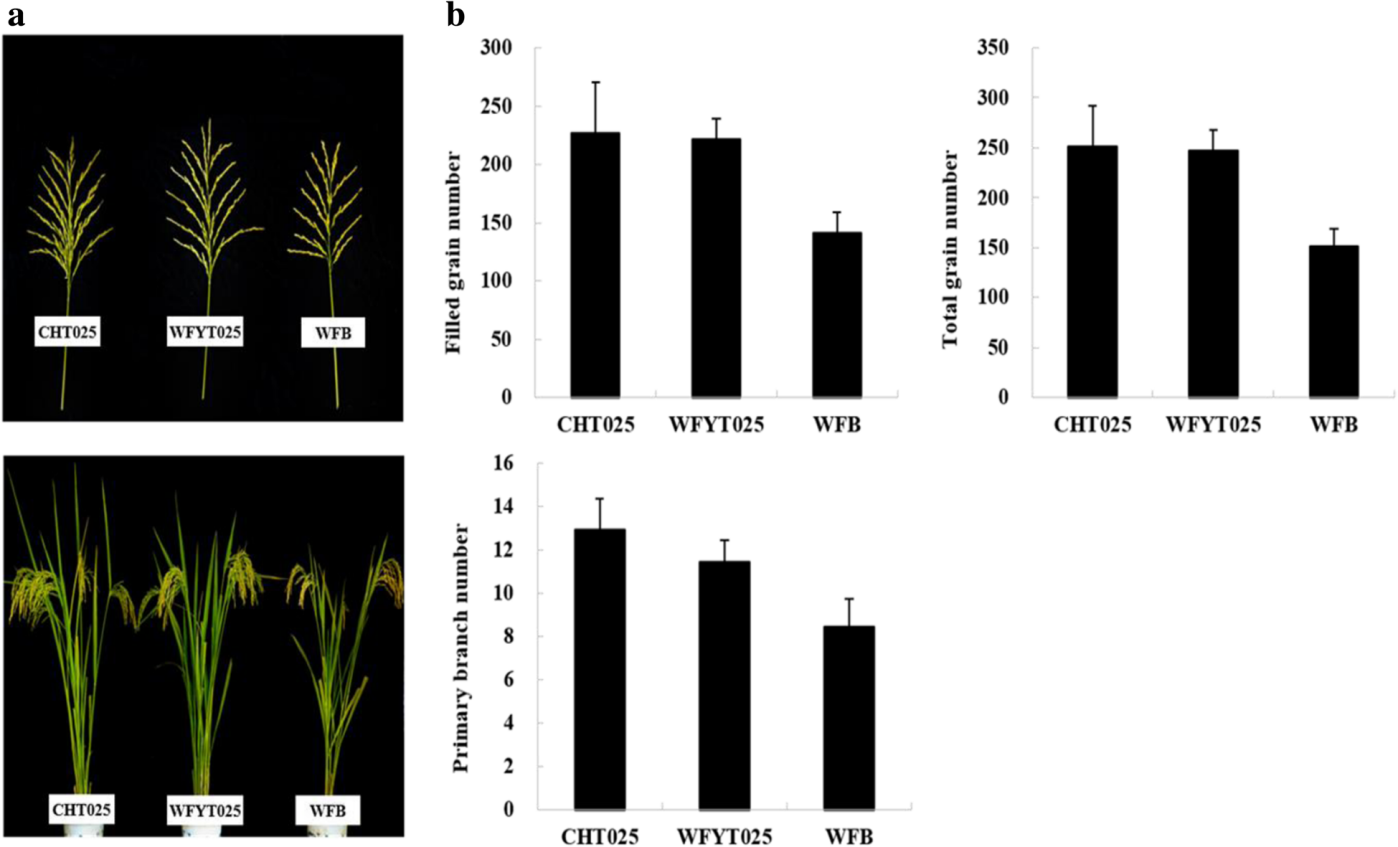
Comparisons of super hybrid WFYT025 combination. **a** The upper panel illustrates the panicles from combination of super hybrid WFYT025. Left, CHT025; middle, WFYT025; right, WFB. The lower panel shows the combination of super hybrid WFYT025. Left, CHT025; middle, WFYT025; right, WFB. **b** Panicle traits of CHT025, WFYT025, and WFB

In this study, we investigated the yield-related traits of WFYT025 and its parents. It was found that the panicles of WFYT025 and its male parent CHT025 were larger than those of the female parent WFB, and their grain number and primary branch number were also higher than those in WFB (Fig. 1a and b). However, no significant differences were observed between WFYT025 and parental line CHT025 for both grain number and primary branch number (Fig. 1b). Mid-parent heterosis (MPH) and higher parent heterosis (HPH) were estimated for the heterosis of panicles. The MPH for all of the traits except the seed setting ratio and tiller remained significant (Table 1). Traits such as primary branch number, secondary branch number, filled grain number, empty grain number and 1000-grain weight were significant for the MPH at *p* < 0.05, while traits such as spike length, total grain number and yield per plant were highly significant at *p* < 0.01. The MPH showed negative effects on the seed setting ratio. Apart from the seed setting ratio and empty grain number, the MPH values for all of the traits varied from 1.16 to 32.32%. In addition, HPH for yield per plant remained highly significant (22.99%) at the *p* < 0.01 level. Further analysis indicated that significant difference for yield per plant was mainly due to the large MPH range for filled grain numbers (20.01%) and 1000-grain weight (6.25%). This implied that compared to the 1000-grain weight, the yield heterosis was more likely to underlay the filled grain number between hybrid WFYT025 and maternal line WFB.

**Table 1:** Phenotypic Analysis of Super Hybrid WFYT025 Combination

Traits	CHT025	WFYT025	WFB	MPH (%)	HPH (%)
Spike length(cm)	23.24 ± 1.75	24.34 ± 0.67	19.61 ± 1.71	13.64^**^	4.74
Primary branch number	12.89 ± 1.45	11.45 ± 1.00	8.46 ± 1.26	7.10^*^	− 11.21
Secondary branch number	49.26 ± 9.66	45.67 ± 5.44	26.51 ± 5.21	20.39^*^	− 7.28
Solid grain number	227.40 ± 42.72	221.43 ± 18.30	141.42 ± 17.60	20.01^*^	− 2.63
Total grain number	251.36 ± 40.39	247.08 ± 21.20	151.21 ± 17.89	22.64^**^	− 1.70
Empty grain number	23.95 ± 6.52	25.65 ± 10.68	9.78 ± 2.49	51.30^*^	7.08
Seed setting ratio (%)	89.65 ± 0.04	89.04 ± 4.29	93.05 ± 1.62	−2.54	−5.16
1000-grain weight (g)	18.64 ± 0.92	22.44 ± 0.63	23.60 ± 0.34	6.25^*^	− 5.15
Tiller	6.8 ± 0.92	8.7 ± 1.25	10.4 ± 2.50	1.16	−16.34
Yield per plant (g)	29.89 ± 7.02	42.8 ± 2.80	34.8 ± 6.89	32.32^**^	22.99^**^

**Significant difference with *p* < 0.01

*Significant difference with *p* < 0.05

### Identification of transcripts by sequencing

**Fig. 2:**
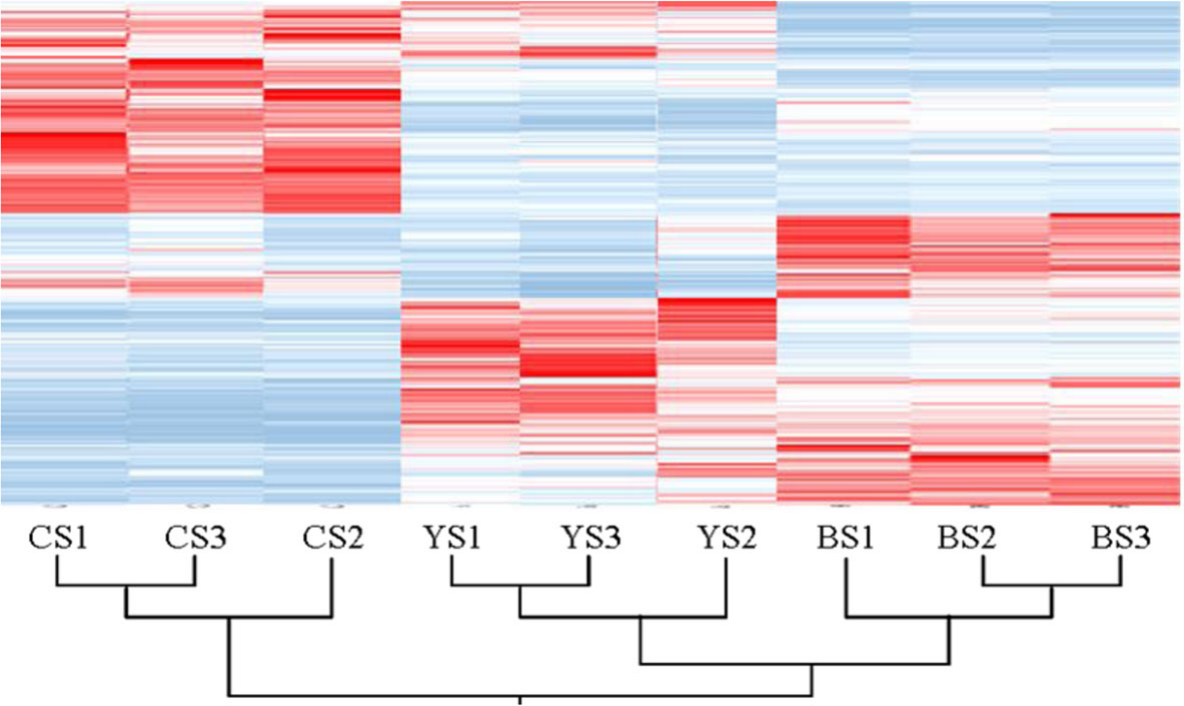
Hierarchical clustering analysis of all gene models based on expression data. Each horizontal line refers to a gene. The color key represents RPKM normalized log2 transformed counts. With the color varied from blue to red, the expression of transcripts are from low to high. CS 1 to 3, YS 1 to 3 and BS 1 to 3 stand for the replicated samples from CHT025, WFYT025, WFB, respectively

A total of 917 million raw reads were generated using the high-throughput Illumina HiSeq 2500 platform. The paired-end sequences with low-quality reads containing adapters were trimmed off. Finally, 87.2 million clean reads were obtained (Table 2). The correlation for the gene expression level from three biological replicates of each line was 0.97 < R^2^ < 0.99. (Additional file 1: Figure S1). We pooled the short reads and aligned them to the Nipponbare reference genome (IRGSP v1.0) to identify the transcripts. Out of 35,679 identified transcripts, 27,917 transcripts were mapped, covering 78.24% of the genome. In addition, the transcriptome profile of WFYT025 was similar to that of its female parent WFB (Fig. 2).

**Table 2:** Number of Mapped Reads

Sample	Total Reads	Mapped Reads	Mapping Ratio (%)
CS	27,507,194	22,157,870	80.55
YS	21,214,278	17,312,734	81.61
BS	38,511,686	31,076,870	80.69
Total	87,233,158	70,547,474	80.95

CS, YS and BS stand for the samples from CHT025, WFYT025, WFB, respectively

### Validation of gene expression by quantitative real-time PCR (qRT-PCR)

**Fig. 3:**
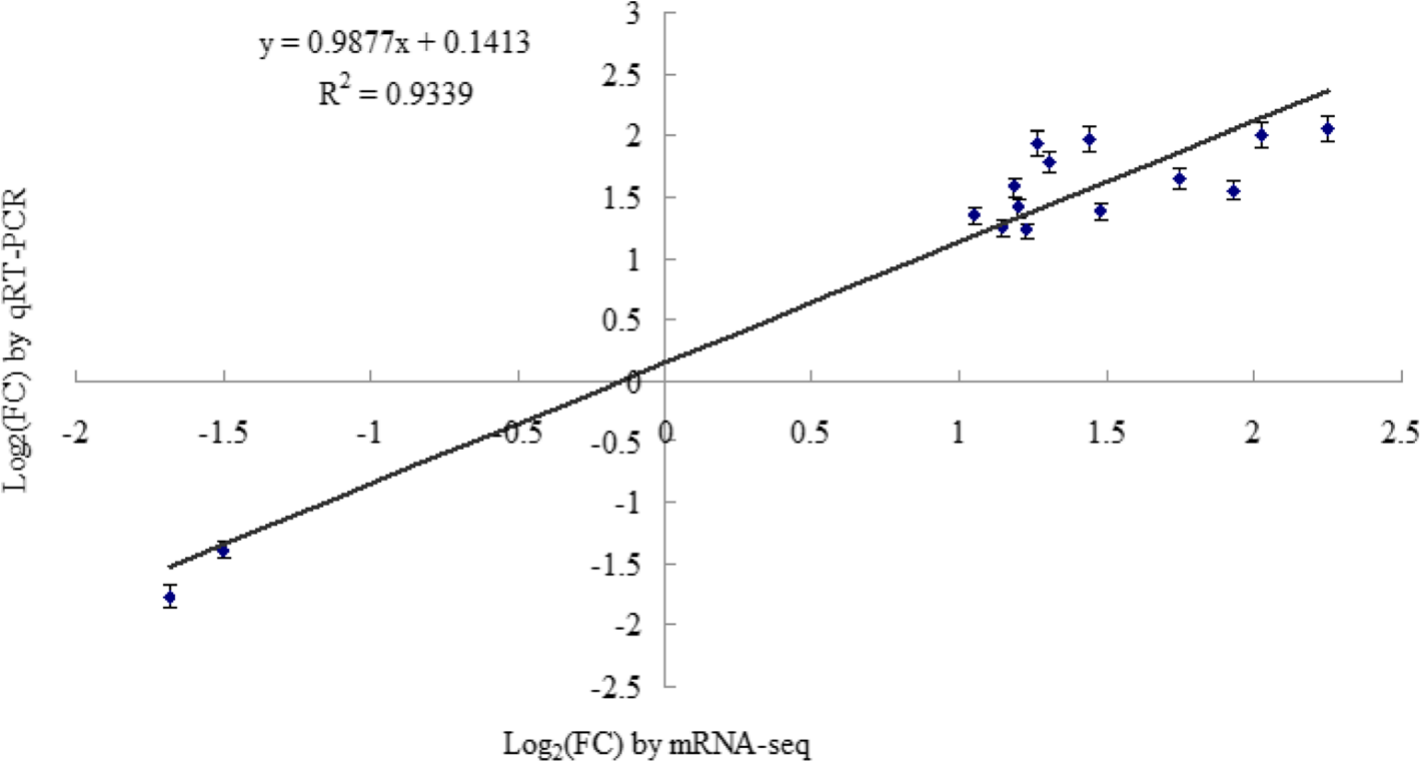
Comparison of the log2 (FC) of 15 randomly selected transcripts using RNA-Seq and qRT-PCR

To validate the results of mRNA sequencing data, the expression of a subset of 15 randomly selected DG_HP_ was determined by qRT-PCR. The list of primer sequences is presented in Additional file 2: Table S1. The results obtained from qRT-PCR and RNA-seq were compared, and expression trends were consistent for all transcripts in both analyses; the correlation coefficient (R^2^) was 0.9339 (Fig. 3).

### Analysis of differentially expressed genes (DEGs)

**Fig. 4:**
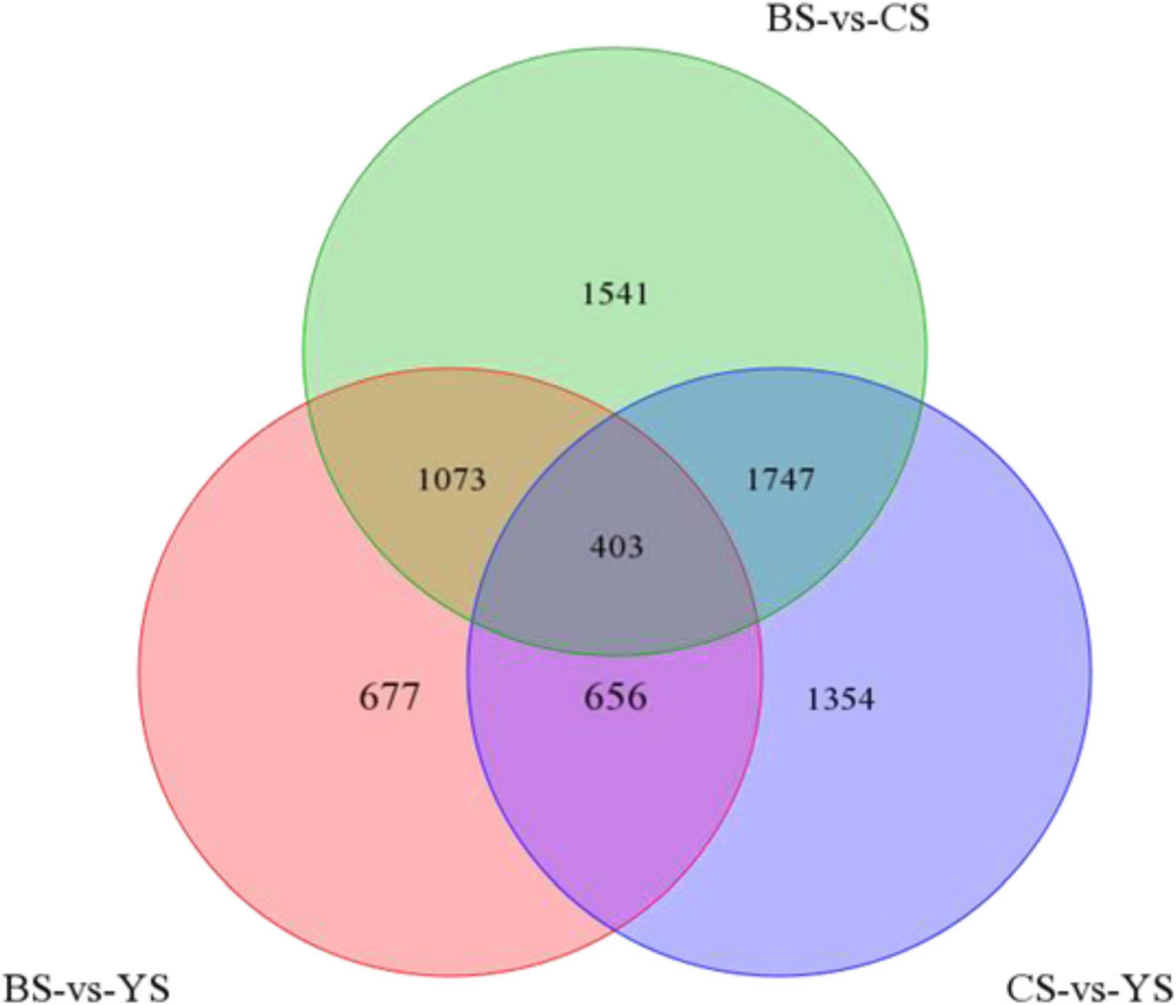
DEGs in super hybrid WFYT025 combination. Venn diagram of DEGs between the hybrid and its parents. CS, YS and BS represent CHT025, WFYT025 and WFB, respectively

We adopted reads per kilobase million reads (FPKM) to measure gene expression levels. Two criteria were considered to identify putative DEGs: (1) the false discovery rate (FDR) should be ≤0.05 and (2) the fold change (FC) should be ≥2. Following these criteria, 4160 DEGs have been identified between paternal line CHT025 and WFYT025. Of these, 2155 DEGs were up-regulated and 2005 were down-regulated. Additionally, 2809 DEGs were identified between maternal line WFB and WFYT025, of which 1463 DEGs were up-regulated and 1346 DEGs were down-regulated (Table 3). For a detailed comparison, the FPKM of all transcripts is presented in Additional file 3: Table S2. DEGs between parents are designated DG_PP_, and DEGs among the hybrid and parents are designated DG_HP_. DG_HP_ may be relevant to heterosis because differences in expression between hybrids and parents should underlie their phenotypic differences. While DG_PP_ only refers to the differences among the two parental lines (Song et al. 2010), there are still 3223 DG_HP_s that overlapped with DG_PP_, which indicates that these DG_PP_ are also associated with heterosis (Fig. 4). In addition, 1059 DG_HP_s were shared between the hybrid and both of its parents.

**Table 3:** Number and Classification of DG_HP_

Pattern	WFYT025 / CHT025	WFYT025 / WFB
Up	2155	1463
Down	2005	1346
Total	4160	2809

### The mode of inheritance for DG_HP_

**Fig. 5:**
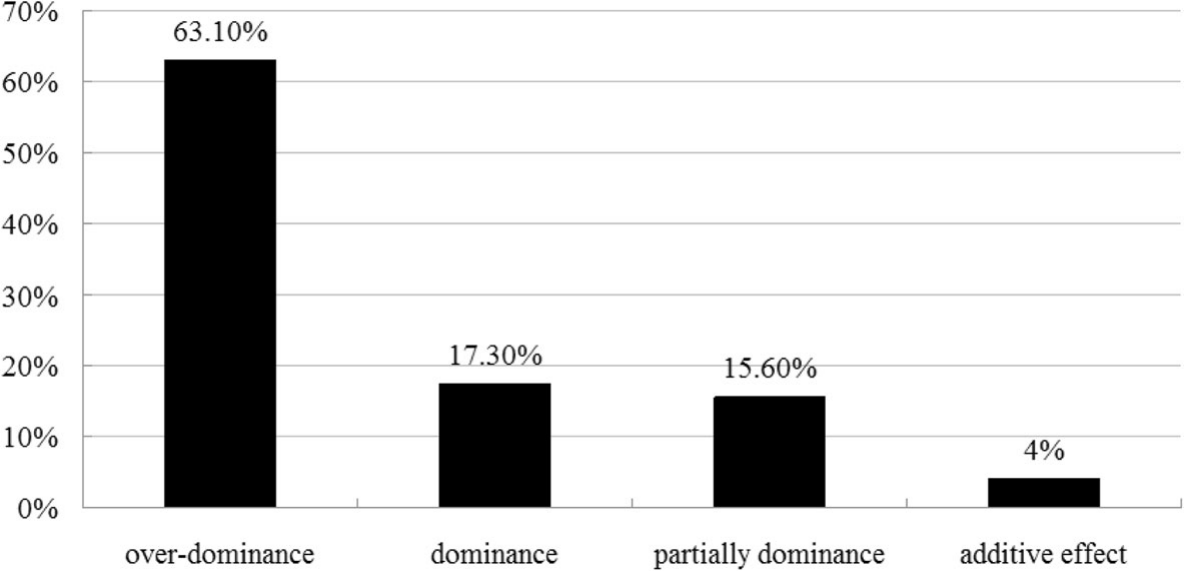
Breakdown of the DGHP according to the dominance ratio Hp. Depending on the principal of Hp = [d] / [a], Hp was classified as either positive or negative

Using the method to evaluate the mode of inheritance, DG_HP_ were classified into four expression patterns: over-dominance (Hp ≤ − 1.2 or Hp > 1.2), dominance (− 1.2 < Hp ≤ − 0.8 or 0.8 < Hp ≤ 1.2), additive effect (− 0.2 < Hp ≤ 0.2), and partial dominance (− 0.8 < Hp ≤ − 0.2 or 0.2 < Hp ≤ 0.8) (Additional file 4: Table S3). As shown in Fig. 5, these data suggested that the over-dominant effect, dominant effect, partially dominant effect and additive effect accounted for 63.1%, 17.3%, 15.6% and 4.0%, respectively.

### Functional classification of DG_HP_ by Gene Ontology (GO)

**Fig. 6:**
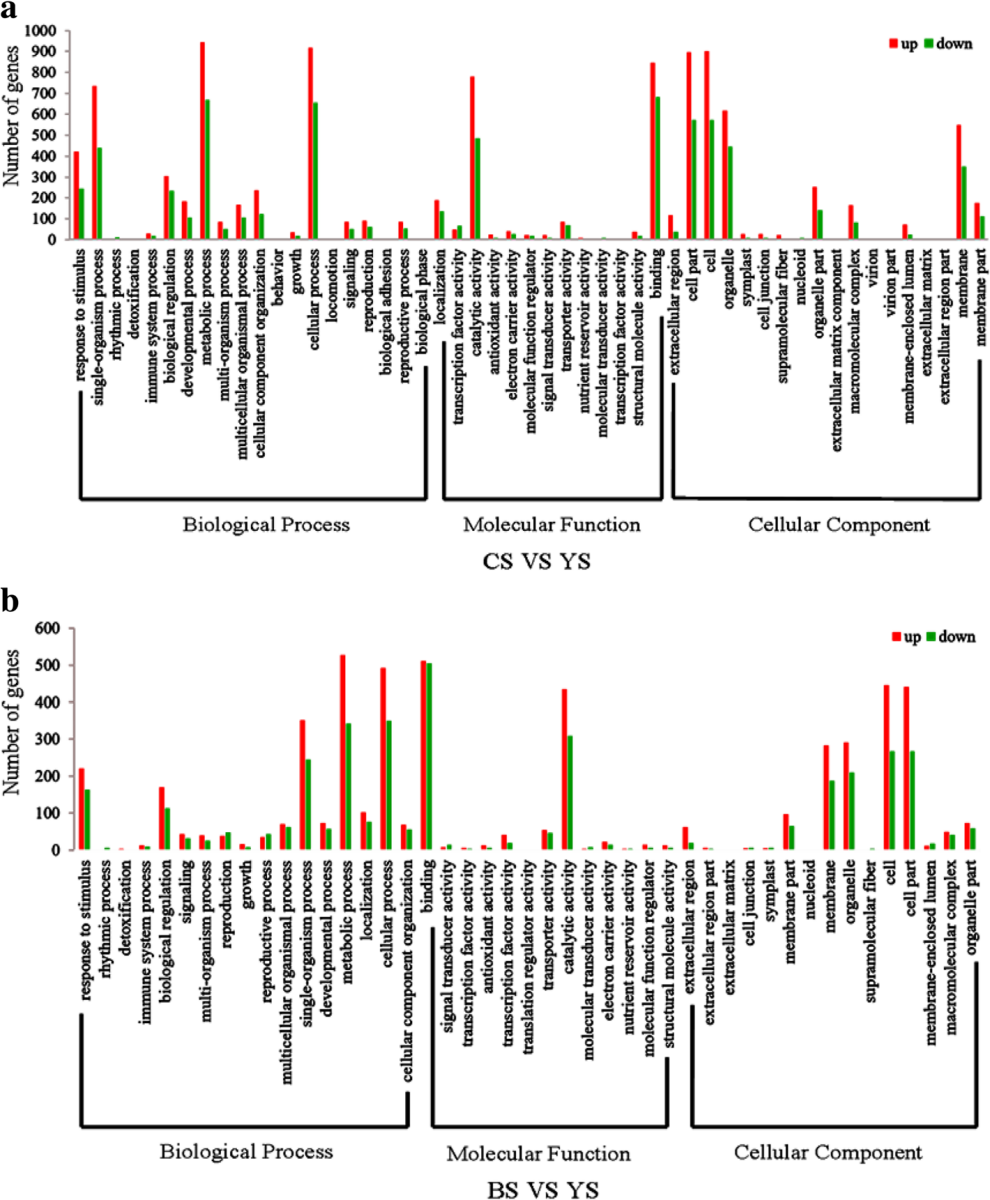
Comparison of Gene Ontology (GO) classifications of DGHP. **a** CS and YS represent CHT025 and WFYT025 respectively. Red column and green column represent up-regulated and down-regulated transcripts respectively. **b** BS and YS represent WFB and WFYT025 respectively. Red column and green column represent up-regulated and down-regulated transcripts respectively

We applied Gene Ontology (GO) to classify the function of the mRNA. Using Web Gene Ontology Annotation Plot (WEGO) software (Ye et al. 2006), we distributed 5910 DG_HP_ into at least one term in the GO molecular function, cellular component, and biological process categories. Further analysis showed that 5910 DG_HP_ were present in 54 functional subcategories at a significance level of *p* < 0.05 (Fig. 6). In the cellular function category, cells and cell parts were mainly divided in the groups. For the molecular function category, DG_HP_ was enriched with binding and catalytic activity. With respect to biological processes, cellular and metabolic processes were highly enriched in DG_HP_. We further analysed the GO terms of DG_HP_ enriched with the biological process subcategories. These GO terms, including response to stimulus, cell proliferation, carbohydrate metabolic process, organ formation, and gibberellin biosynthetic process, may underlie heterosis in the young panicle of WFYT025 (Tables 4 and 5).

**Table 4:** Significant GO Terms of DG_HP_ Between CS and YS in the Biological Process Category

GO ID	Description	*p*-value
GO:0042221	Response to chemical	0.000000
GO:0010035	Response to inorganic substance	0.000000
GO:0008283	Cell proliferation	0.000000
GO:0006260	DNA replication	0.000000
GO:0005975	Carbohydrate metabolic process	0.000004
GO:0006629	Lipid metabolic process	0.000088
GO:0009725	Response to hormone	0.000276
GO:0044550	Secondary metabolite biosynthetic process	0.000291
GO:0000281	Mitotic cytokinesis	0.000378
GO:0061640	Cytoskeleton-dependent cytokinesis	0.000378
GO:0051301	Cell division	0.001546
GO:0019344	Cysteine biosynthetic process	0.001784

**Table 5:** Significant GO Terms of DG_HP_ Between BS and YS in the Biological Process Category

GO ID	Description	*p*-value
GO:0006950	Response to stress	0.000012
GO:0050896	Response to stimulus	0.000047
GO:0048645	Organ formation	0.006863
GO:0071265	L-methionine biosynthetic process	0.008974
GO:0009686	Gibberellin biosynthetic process	0.009689
GO:0010160	Formation of organ boundary	0.011830
GO:0003156	Regulation of organ formation	0.016135
GO:0045596	Negative regulation of cell differentiation	0.016135
GO:0048497	Maintenance of floral organ identity	0.016135
GO:0010077	Maintenance of inflorescence meristem identity	0.017878
GO:2000027	Regulation of organ morphogenesis	0.030664
GO:0048586	Regulation of long-day photoperiodism, flowering	0.034206
GO:2000028	Regulation of photoperiodism, flowering	0.044460

### DG_HP_ mapping Kyoto Encyclopedia of Genes and Genomes (KEGG) pathway

**Fig. 7:**
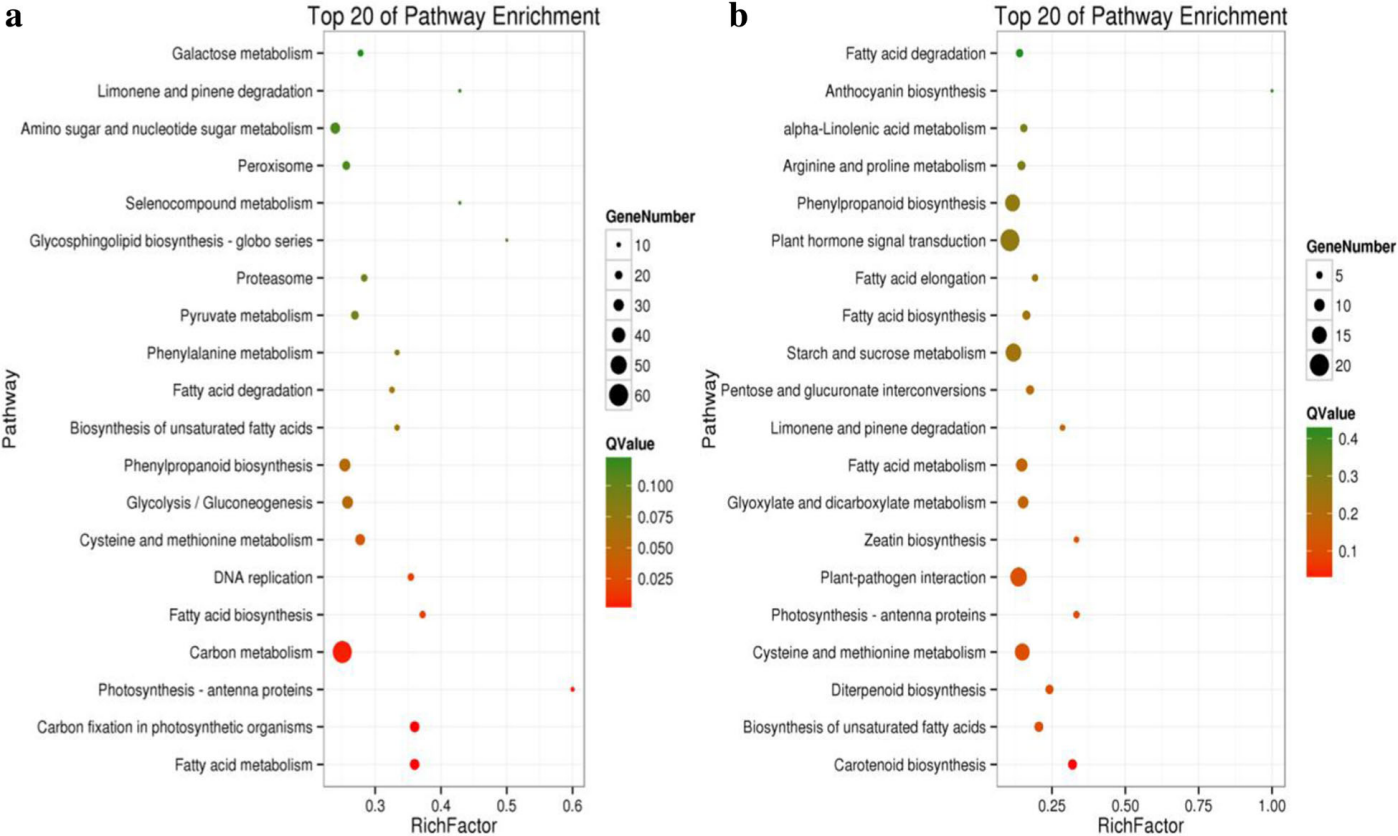
KEGG pathway assignments of DGHP. **a** KEGG analysis of DGHP between CHT025 and WFYT025. **b** KEGG analysis of DGHP between WFB and WFYT025. Both (**a**) and (**b**) showed the top 20 most represented categories and the number of transcripts predicted to belong to each category

For the identification of metabolic pathways in which DG_HP_ were involved and enriched, the Kyoto Encyclopedia of Genes and Genomes pathway database was used. In total, 118 pathways were identified in 613 DG_HP_ (between paternal line CHT025 and hybrid line WFYT025). The top 20 most enriched pathways mainly covered carbon fixation in photosynthetic organisms, DNA replication, fatty acid biosynthesis and metabolism, and phenylpropanoid biosynthesis (Fig. 7a). In contrast, 268 DG_HP_ between maternal line WFB and WFYT025 were classified into 107 pathways, and the top 20 most enriched pathways were mainly concentrated in plant hormone signal transduction, carotenoid biosynthesis, diterpenoid biosynthesis, zeatin biosynthesis, and cysteine and methionine metabolism with a significance level of *p* < 0.05 (Fig. 7b). This suggests that the considerable differences in young panicles between WFB and WFYT025 may be related to hormone regulation.

### Comparison of DG_HP_ with grain yield-related genes (QTLs)

We were able to map the DG_HP_ that were significant in the KEGG analysis (*P* < 0.05) between WFYT025 and WFB for the QTLs associated with grain yield in the rice genome (http://www.gramene.org). As shown in Table 6, a total of 36 transcripts were mapped in the interval of 22 yield-related QTLs, including 15 grain number QTLs, 6 1000-grain weight QTLs and 1 yield per plant QTL. Most genes shared the same location with one yield-related QTL. However, Os03g0856700 corresponded to *qGP3–1* for grain number and *qSNP-3b* for spikelet number per panicle. Os04g0229100 was mapped to the same loci as *qGwt4a* for 1000-grain weight and *qSNP-4a for* spikelet number per panicle, while Os04g0578400 and Os04g0608300 shared the same chromosome segment with *qGPP-4* for grain number per panicle and *qSNP4–1* for spikelet number per panicle.

**Table 6:** Significant differentially Expressed Transcripts Mapped in each of the QTL Regions

Trait	QTL	Chr	Intervel	DG_HP_
GPP	*qGP-1a*	1	RM1-R753	Os01G0135700, Os01G0150800
NGP	*qNG-1*	1	RG374-RG394	Os01G0788400
NSP	*qSSBP1–1*	1	C86-C2340	Os01G0846300
GW	*qgw362*	2	C1445-C560	Os02G0697400, Os02G0771600
GPP	*qGP3–1*	3	G249-RG418	Os03G0760200,Os03G0762400,Os03G0797800, Os03G0856700
SNPP	*qSNP-3b*	3	RM227-RM85	Os03G0856700
GW	*qGW3.1*	3	RZ672-RZ474	Os03G0423300, Os03G0645900
GW	*qGwt4a*	4	RG788-RG190	Os04G0229100
SNPP	*qSNP-4a*	4	RM401-RM335	Os04G0229100,Os04G0474800,Os04G0486950,
GPP	*qGPP-4*	4	RZ569-RZ565	Os04G0492800,Os04G0498700,Os04G0518100, Os04G0522500 Os04G0535600,Os04G0556500,Os04G0565200, Os04G0578400,Os04G0608300,Os04G0611700, Os04G0611800,Os04G0618700
SNPP	*qSNP4–1*	4	RM303-RM255	Os04G0578400,Os04G0608300
GW	*qKw5*	5	RG182-RG13	Os05G0374200, Os05G0380900
SD	*qSD-15*	5	RG13-RG346	Os05G0475400,Os05G0551700,Os05G0408900
GW	*qGw-6*	6	C235-G294	Os06G0347100, Os06G0486900
SP	*qSP6–1*	6	RG138-RZ398	Os06G0185100
GPP	*qGP-6*	6	RZ667-RG424	Os06G0347100
SSP	*qSPN-6*	6	C236-G294	Os06G0486900
GW	*qGw7*	7	R1440-RG128	Os07G0154100, Os07G0155600
YPP	*yd7a*	7	R1440-RG128	Os07G0154100, Os07G0155600
GPP	*qGP-7a*	7	R1440-RG128	Os07G0154100
SSD	*qSSD-10*	10	RG257-RZ583	Os10G0419400,Os10G0422200,Os10G0430200, Os10G0472900
NFPB	*qNFPB-11*	11	RM286-RM332	Os11G0141400, Os11G0152700

## Discussion

Though heterosis has been extensively exploited in plant breeding and plays an important role in agriculture, the molecular and genetic mechanisms underlying this phenomenon remain poorly understood. Differential gene expression between a hybrid and its parents may be associated with heterosis (He et al. 2010; Kim et al. 2013; Zhang et al. 2008). Here, we investigated the relationship between transcriptional profiles and heterosis in super hybrid rice WFYT025 by RNA-Seq.

### Comparative analysis of DG_HP_

Using RNA-Seq analysis, 872 million high-quality paired-end reads of 150 bp were generated from the panicles of WFYT025 and its parental lines at the panicle differentiation stage, and 27,917 annotated transcripts were identified. Of these transcripts, 4160 DG_HP_ between hybrid WFYT025 and paternal line CHT025 and 2809 DG_HP_ between hybrid WFYT025 and maternal line WFB were identified.

The filled grain number heterosis exhibited significant differences between WFYT025 and WFB; however, there were no significant differences between WFYT025 and CHT025 (Fig. 1b, Table 1). Therefore, the results suggest that the expression of DG_HP_ between WFYT025 and WFB at the young panicle development stage may play an important role in grain number heterosis compared to that between WFYT025 and CHT025. Therefore, focusing on the expression of DG_HP_ between WFB and WFYT025 might find an association between DG_HP_ and heterosis for filled grain number.

### The genetic basis of heterosis

We have been able to identify a number of DG_HP_s underlying grain number between hybrid WFYT025 and maternal line WFB, confirming the suggestion that heterosis is a polygenic phenomenon (Kusterer et al. 2007; Bian et al. 2011). Among the DG_HP_, 17.3% had a dominant effect, 15.6% had a partial dominant effect, 4% had an additive effect and the remaining 63.1% had an over-dominant effect. Thus, over-dominance was the major contributor to the heterosis of WFYT025.

Meanwhile, the expression differences of cloned yield trait genes have been investigated between the hybrid and its parents. Of the 143 genes related to grain yield traits, 11 genes, accounting for 7.7%, showed over-dominance; 12 genes, accounting for 8.3%, showed dominance; 71 genes, accounting for 49.6%, showed partial dominance; and 49 genes, accounting for 34.4%, showed partial dominance (Additional file 5: Table S4).

### The role of hormone signal transduction in heterosis

It is well known that hormones act as signalling molecules in plants and can regulate physiological responses. Transcriptome analysis has uncovered many DG_HP_s that are involved in the phytohormone response in young panicle tissue. For example, mRNA levels of Os12g0586100 encoding SNF1-related protein kinase2 (SnRK2), whose autophosphorylation is required for kinase activity towards downstream targets, were expressed poorly in WFYT025 compared to its parents. In addition, type-2C protein phosphatase (PP2C, a negative regulator) (Os01g0846300, Os05g0572700, Os01g0656200 and Os03g0268600) was up-regulated, and a similar observation was also reported by Merlot et al. (2001) and Zhai et al. (2013). These results are consistent with the negative-feedback regulatory mechanism in ABA signal transduction.

Moreover, transcripts involved in the gibberellin (GA) biosynthesis pathway were also differentially expressed between the hybrid and its two parents, in this study. GAs are a large family of diterpenoid compounds, some of which are bioactive growth regulators that control flower development (Cowling et al. 1998). GAs are involved in the transformation of vegetative reproduction to reproductive growth (Poethig 1990; Evans and Poethig 1995). *OsGA20ox1* (Os03g0856700) encodes a GA20 oxidase, which is the key enzyme that catalyses the penultimate step reaction of gibberellin biosynthesis and enhances the grain number of rice by increasing the cytokinin activity in the rice panicle meristem (Wu et al. 2016). In this study, we observed that the expression level of *OsGA20ox1* in WFYT025 is up-regulated 2-fold higher than in WFB and showed over-dominance (Additional file 5: Table S4). This suggested that WFYT025 may possess strong potential for gibberellin biosynthesis compared to maternal line WFB, which promoted the amount of spikelet primordium in hybrid line WFYT025.

### The significant DG_HP_ related to grain yield QTLs

We compared the significantly enriched DG_HP_ to grain yield QTLs. As shown in Table 6, among the DG_HP_-correlated QTLs, many QTLs were well characterized, including those for grain per panicle (e.g., q*GP-1a* (Yu et al. 1997), *qNG-1* (Lin et al. 1996), *qGP3–1* (Li et al. 2001), *qGPP-4* (Xiao et al. 1996), *qGP-6* (Hua et al. 2002), *qGP-7a* (Li et al. 2000)); number of spikelets on secondary branches per panicle (e.g., *qSSBP1–1* (Cui et al., 2002)); spikelet number per panicle (e.g., *qSNP-3b* (Xu et al. 2001)*, qSNP-4a* (Mei et al. 2006)*, qSNP4–1* (Takai et al. 2005)*, qSP6–1* (Zhuang et al. 2001)*, qSNP-6* (He et al. 2001)*, qNFPB-11* (Yamagishi et al. 2004)); spikelet density (e.g., *qSD-15* (Li et al. 1998) and *qSSD-10* (Xiao et al. 1996)); 1000-grain weight (e.g., *qgw362* (Ishimaru 2003), *qGW3.1* (Thomson et al. 2003), *qGwt4a* (Lin et al. 1995), *qKw5* (Li et al. 1997), *qGw-6* (Lu et al. 1996), and *qGw7* (Li et al. 2000)); and yield per plant (e.g., *yd7a* (Li et al. 2000)).

The potential association between DG_HP_ and QTLs was also suggested within many QTL regions, including putative protein phosphatase 2C (Os01g0846300) with *qSSBP1–1* for the number of spikelets on secondary branches per panicle and putative transketolase (Os05g0408900) with *qSD-15* for spikelet density. Interestingly, *OsGA20ox1* (Os03g0856700), which is related to gibberellin biosynthesis, is located in both *qGP3–1* for the number of grains per panicle and *qSNP-3b* for the spikelet number per panicle. Putative fatty acid hydroxylase (Os04g0578400), which is involved in carotenoid biosynthesis, and OsSAUR20-Auxin-responsive SAUR gene family member (Os04g0608300) was shared in both *qSNP4–1* for spikelet number per panicle and *qGPP-4* for number of grains per panicle. Except for a small number of cloned genes, such as Os01g0788400, Os02g0697400, Os02g0771600, *OsGA20ox1* (Os03g0856700), Os03g0760200, Os03g0645900, Os04g0474800, Os04g0522500*,* Os04g0556500, Os05g0380900, Os07g0154100, and Os07g0155600, the remaining genes(including Os01g0846300, Os05g0408900, Os04g0578400 and Os04g0608300), which have been located in grain yield QTLs (including grain number, 1000-grain weight, and yield), were not cloned. Studying the function of these candidate transcripts in these QTL regions may increase the knowledge of the molecular mechanisms underlying heterosis.

### Transcription factors probably underlying heterosis

Since transcripts are always under different levels of regulation, such as transcription and splicing through genetic or epigenetic mechanisms, the detailed sequence comparisons and validations for different alleles of annotated DG_HP_ are not suitable to display in this current report. Transcription factors (TFs) are certainly one of the causes of gene expression fluctuations. In this study, we indeed found that 51 TFs showed significant differential expression in the hybrid compared with the maternal line (Additional file 6: Table S5). It is a coincidence that a previous study also proposed that altered gene expression caused by interactions between transcription factor allelic promoter regions in hybrids was one reasonable mechanism underlying heterosis in rice (Zhang et al. 2008).

Furthermore, among the 51 TFs, we found that *LAX1*, which is the main regulator involved in the formation of axillary bud primordium in rice, is overrepresented in the hybrid (Komatsu et al. 2003). MADS-box 55 (*MADS50*) was up-regulated significantly, and MADS-box 56 (*MADS56*) was down-regulated in the hybrid compared to the maternal line (Additional file 6: Table S5). This is consistent with a previous study that suggests that *OsMADS50* and *OsMADS56* function antagonistically in regulating LD-dependent flowering (Ryu et al. 2009). Certainly, except for 21 reported TFs, the remaining 30 novel TFs might play an important role in the young panicle and grain number heterosis.

## Conclusions

In this study, we systematically investigated the transcriptome profiles from super-hybrid rice WFYT025 combinations for young panicles at the panicle differentiation stage by deep high-quality sequencing. We obtained a large amount of DG_HP_ and found that the over-dominance effect is the main mode of inheritance for DG_HP_. Comparing the significantly enriched DG_HP_ (*P* < 0.05) between WFYT025 and WFB with QTLs in response to grain number, we found some candidate transcripts that may contribute to the increase in grain yield. Exploring these candidate transcripts will provide new opportunities for revealing the heterosis of grain yield.

## Methods

### Plant materials and growth conditions

The hybrid WFYT025 along with its parental lines Changhui T025 (CHT025) and Wufeng B (WFB) were planted in the experimental field of Jiangxi Agricultural University. WFYT025 is a super-hybrid rice combination derived from the cross between female parent WFB and male parent CHT025. WFYT025 and the two parents were sown at the experimental plot in Jiangxi Agricultural University in a completely randomized block design with three replications in autumn 2016. Each plot consisted of 50 rows, with each row consisting of 10 plants, each separated from its neighbour by 20 cm. Crop management followed normal procedures for rice. These three lines were selected in this study to measure phenotypic traits and conduct transcriptome analyses. At maturity time, panicles were selected with ten replicates for the estimation of heterosis. The young panicles at the differentiation stage were collected and stored at − 80 °C for RNA-Seq analysis, and each sample had at least three biological replications to minimize systematic errors.

### Panicle heterosis measurements

To determine 1000-grain weight, panicles were dried in an oven at 42 °C for 1 week. Panicle length, primary branch, secondary branch, number of filled grains and total grain number were measured manually. Mid-parent heterosis (MPH) and higher parent heterosis (HPH) were calculated for these traits according to the following formulas: MPH = (F_1_ − MP) / MP and HPH = (F_1_ − BP) / BP, where F_1_ is the performance of the hybrid, MP is the average performance of the two parents and BP is the performance of better parents. Hypothesis testing was performed using a *t*-test.

### RNA extraction, cDNA library preparation and sequencing

Total RNA was extracted from rice panicles using Trizol reagent (Invitrogen, Carlsbad, CA, USA) and purified using an RNeasy Plant Mini Kit (Qiagen, Valencia, CA, USA) according to the manufacturer’s instructions. The quality and integrity of RNA were tested using an Agilent Bioanalyzer 2100 system (Agilent, Santa Clara, CA, USA); RNA Integrity Number (RIN) values were greater than 8.5 for all samples. After total RNA extraction, eukaryotic mRNA was enriched by Oligo (dT) beads, while prokaryotic mRNA was enriched by removing rRNA using the Ribo-Zero TM Magnetic Kit (Epicentre). Then, the enriched mRNA was fragmented into 200-bp segments using fragmentation buffer and reverse transcribed into cDNA with random primers. Second-strand cDNA synthesis was subsequently performed using DNA polymerase I, RNase H, dNTP and buffer. Then, the cDNA fragments were purified with QIAquick PCR extraction kit, end repaired, poly (A) added, and ligated to Illumina sequencing adapters. The ligation product size was selected by agarose gel electrophoresis, PCR amplified, and sequenced with 100 cycles of paired-end sequencing (2 × 150 bp) using Illumina HiSeq TM 2500 by Gene Denovo Biotechnology Co. (Guangzhou, China). The processing of fluorescent images into sequences, base-calling and quality value calculations were performed using the Illumina data processing pipeline (version 1.8). The sequence reads were submitted to the NCBI Sequence Read Archive (SRA, http://www.ncbi.nlm.nih.gov/sra) under the accession number SRP127997.

### Identification of differentially expressed mRNAs

Raw reads generated from high-throughput sequencing were treated as follows. First, to remove adapters that were added for reverse transcription and sequencing, sequences with too many unknown bases (>10%) and low-quality bases (>50% of the bases with a quality score ≤ 20) were removed. The reads mapped to the ribosome RNA (rRNA) database were removed with the read alignment tool Bowtie 2 (Langmead and Salzberg 2012). The remaining reads of each sample were then mapped to the Nipponbare reference genome (IRGSP build 1.0) by TopHat2 (version 2.0.3.12) (Kim et al. 2013). The parameters for alignment were set as follows: 1) the maximum read mismatch should be 2; 2) the distance between mate-pair reads should be 50 bp; 3) the error of distance between mate-pair reads should be ±80 bp. Differential expression was estimated and tested using the software package edgeR (R version: 2.14, edge R version: 2.3.52) (Robinson et al. 2010). We quantified gene expression levels in terms of fragments per kb for a million reads (FPKM) (Mortazavi et al. 2008), calculated the false discovery rate (FDR), and estimated the fold change (FC) and log _2_ values of FC. Transcripts that exhibited an FDR ≤ 0.05 and an estimated absolute log_2_(FC) ≥ 1 were considered to be significantly differentially expressed.

### The mode of inheritance analysis

For statistical analysis, the analysis of variance (ANOVA) was usually by the model: y = u + (GA) + (GD) + (SR) + e, where y is the acquired gene expression, u is the overall mean, GA is the additive effect, GD is the dominant effect, SR is the replication effect, and e is the residual error (Lynch and Walsh 1998). Hp = [d]/[a], referred to as the dominance ratio or potency (where [a] and [d] represent GA and GD, respectively), was also calculated to measure the non-additivity of the F_1_ hybrid relative to its parents (Griffing 1990). Considering gene expression levels as quantitative traits, we adopted traditional quantitative genetic parameters, such as composite additive effect [a] and composite dominance effect [d], to estimate our expression profile. DG_HP_ were classified according to the dominance ratio Hp (= [d]/[a]), based on 99.8% confidence intervals constructed for [*d*] - [*a*] ([*d*] > 0) and [*d*] + [*a*] ([*d*] < 0). According to the value of Hp (=[d]/[a]), we considered that these genes belonged to partial dominance (− 0.8 < Hp ≤ − 0.2 or 0.2 < Hp ≤ 0.8), over-dominance (Hp ≤ − 1.2 or Hp > 1.2), dominance (− 1.2 < Hp ≤ − 0.8 or 0.8 < Hp ≤ 1.2) and additive effect (− 0.2 < Hp ≤ 0.2) (Stuber et al. 1987, Bian et al. 2011).

### Cluster analysis

Cluster analysis of all annotated transcripts from the hybrid and its parents was performed. The FPKM-normalized expression counts for each transcript were clustered with the software Cluster 3.0, and the results were visualized using Treeview (Eisen et al. 1998).

### Real-time quantitative PCR

The expression of genes with differential expression (DEGs) and results of RNA sequencing were validated by quantitative real-time PCR. Total RNA from nine samples (including three biological replicates) was extracted using the Prime Script™ RT reagent Kit with gDNA Eraser according to the manufacturer’s instructions. SYBR-based qRT-PCR reactions (SYBR Green I, Osaka, Japan) were performed on an ABI VIIA@7 using the following thermal cycling conditions: 50 °C for 2 min; 95 °C for 5 min followed by 40 cycles at 95 °C for 15 s and 60 °C for 34 s. All qRT-PCR reactions were performed in triplicate samples, and the results were analysed with the system’s relative quantification software (ver. 1.5) based on the (ΔΔCT) method. The detection of the threshold cycle for each reaction was normalized against the expression level of the rice Actin1 gene with the primer sequences 5′-TGGCATCTCTCAGCACATTCC-3′ and 5′-TGCACAATGGATGGGTCAGA-3′.

## Additional files

Additional file 1: **Figure S1.** Scatterplots comparing gene expression scores from biological replicates of WFYT025 and its parents. CS 1-3, YS 1-3, and BS 1-3 denote biological replicates from CHT025, WFYT025 and WFB, respectively. (DOC 142 kb)

Additional file 2: **Table S1.** Primer sequences for qRT-PCR expression analysis. (XLS 17 kb)

Additional file 3: **Table S2.** The FPKM of all transcripts. (XLS 113176 kb)

Additional file 4: **Table S3.** Classification of DG_HP_ based on the dominance ratio H_P. (XLS 526 kb)_

Additional file 5: **Table S4.** The mode of inheritance of cloned genes. (XLS 45 kb)

Additional file 6: **Table S5.** The DG_HP_ of all transcription factors between WFYT025 and WFB. (XLS 63 kb)

## Abbreviations

ABA: Abscisic Acid
DEGs: The Genes with Different Expression
DG_HP_: The Genes with Different Expression Between the Hybrid and Parents
DG_PP_: The Genes with Different Expression Between Paternal Line and Maternal Line
FC: Fold Change
FDR: False Discovery Rate
FPKMs: Fragments Per kb for a Million Reads
GA: Gibberellins
GO: Gene Ontology
GPP: Grain per Panicle
GPP: Grains per Panicle
GW: Grain Weight
HPH: Higher Parent Heterosis
KEGG: Kyoto Encyclopedia of Genes and Genomes
MPH: Mid-Parent Heterosis
NFPB: Number of Florets per Branch
NGP: Number of Grains per Panicle
NSP: Number of Spikelets on Secondary Branches Per Panicle
PP2C: Type-2C Protein Phosphatase
qRT-PCR: Quantitative Real-Time Polymerase Chain Reaction
QTL: Quantitative Trait Locus
RNA-seq: RNA Sequencing Technology
SD: Spikelet Density
SNPP: Spikelet Number per Panicle
SnRK2: SNF1-Related Protein Kinase2
SP: Spikelet Number per Panicle
SSD: Spikelets Setting Density
SSP: Spikelet Number per Panicle
TFs: Transcription Factors
WEGO: Web Gene Ontology Annotation Plot Software
YPP: Yield per Plant
