# Supplementary figures and images for: Genetic analysis for the grain number heterosis of a super-hybrid rice WFYT025 combination using RNA-Seq
Source: Rice (N Y). 2018 Jun 15;11:37. doi: 10.1186/s12284-018-0229-y (PMC6003258; doi:10.1186/s12284-018-0229-y)

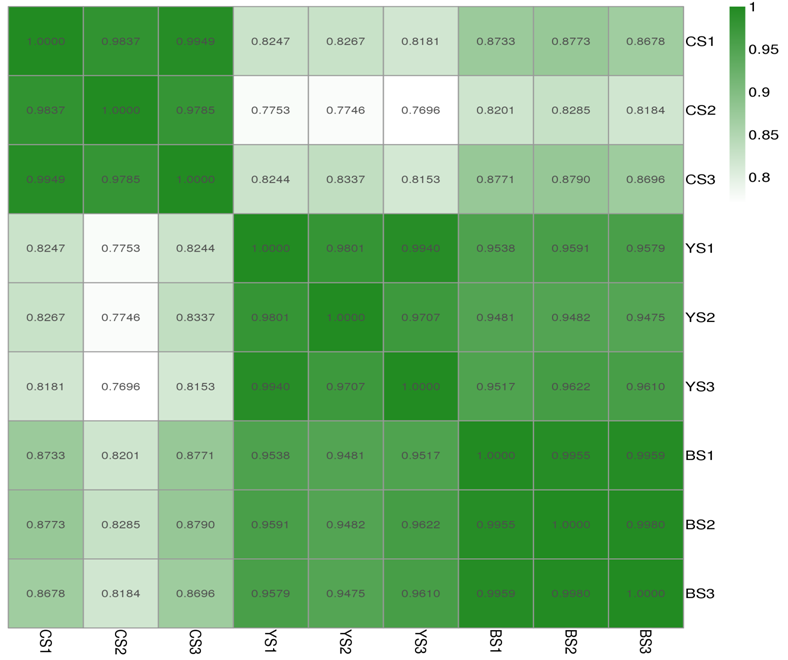

Supplement: Supplementary file 1 — Figure S1. Scatterplots comparing gene expression scores from biological replicates of WFYT025 and its parents. CS 1-3, YS 1-3, and BS 1-3 denote biological replicates from CHT025, WFYT025 and WFB, respectively. (DOC 142 kb) [file 12284_2018_229_MOESM1_ESM.doc]
